# Endothelial protease-activated receptor 4: impotent or important?

**DOI:** 10.3389/fcvm.2025.1541879

**Published:** 2025-01-28

**Authors:** Rahul Rajala, Courtney T. Griffin

**Affiliations:** ^1^ Cardiovascular Biology Research Program, Oklahoma Medical Research Foundation, Oklahoma City, OK, United States; ^2^ Department of Cell Biology, University of Oklahoma Health Sciences Center, Oklahoma City, OK, United States; ^3^ Harold Hamm Diabetes Center, University of Oklahoma Health Sciences Center, Oklahoma City, OK, United States

**Keywords:** PAR4, protease, vascular biology, G protein-coupled receptor (GPCR), endothelial cell, thrombin, signal transduction

## Abstract

The protease thrombin, which increases its levels with various pathologies, can signal through the G protein-coupled receptors protease-activated receptors 1 and 4 (PAR1/PAR4). PAR1 is a high-affinity receptor for thrombin, whereas PAR4 is a low-affinity receptor. Finding functions for PAR4 in endothelial cells (ECs) has been an elusive goal over the last two decades. Several studies have demonstrated a lack of functionality for PAR4 in ECs, with many claiming that PAR4 function is confined mostly to platelets. A recent study from our lab identified low expressing but functional PAR4 in hepatic ECs *in vivo.* We also found that PAR4 likely has a higher signaling potency than PAR1. Given this potency, ECs seem to limit PAR4 signaling except for extreme cases. As a result, we claim PAR4 is not an impotent receptor because it is low expressing, but rather PAR4 is low expressing because it is a very potent receptor. Since we have finally shown PAR4 to be present and functional on ECs *in vivo*, it is important to outline why such controversy arose over the last two decades and, more importantly, why the receptor was undervalued on ECs. This timely review aims to inspire investigators in the field of vascular biology to study the regulatory aspect of endothelial PAR4 and its relationship with the more highly expressed PAR1.

## Introduction

The vascular endothelium, which is composed of the innermost lining of cells in blood vessels, covers a surface area of 270–720 square meters in humans (1, 2). Endothelial cells (ECs) play numerous roles in regulating tissue structure (3) and function (4) by impacting inflammation (5), permeability (6), and trafficking of proteins and nutrients (6). Given these roles, it is no surprise that dysfunctional ECs can contribute to pathological insults (7). One of these insults is a *proteolytic storm*; like a cytokine storm, it is a sudden and rapid rise of protease activity. In the vasculature, this can lead to disseminated intravascular coagulopathy (8), sepsis (9), and some cancers (10). Given that proteases and their inhibitors constitute over 2% of the genes in the human genome (11), understanding how these proteases signal, particularly in ECs, is critical to understanding disease progression.

**Figure 1:**
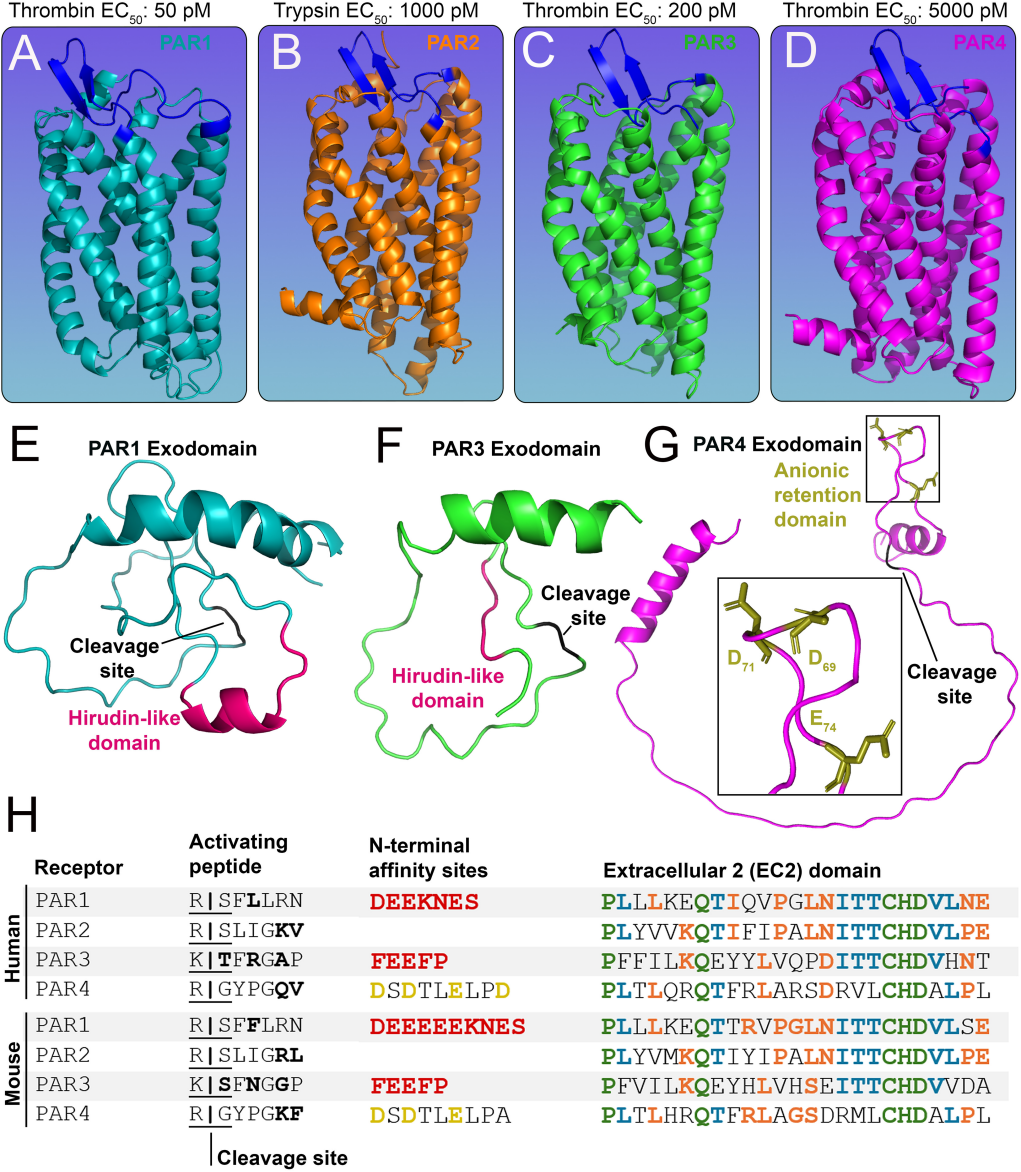
Structures of the PAR family of receptors. **(A-D)** *In silico* models of murine **(A)** PAR1 (cyan) **(B)** PAR2 (orange) **(C)** PAR3 (green) and **(D)** PAR4 (pink) with EC_50_ values for predominant activating proteases shown above. The extracellular 2 (ECL2) domain of each receptor is shown in blue. **(E-G)** The N-terminal exodomain of **(E)** PAR1 and **(F)** PAR3 with a red hirudin-like domain (HLD) and a black cleavage site. The N-terminal exodomain of **(G)** PAR4 shows the anionic retention domain in gold and the cleavage site in black. **(H)** Tables of human and mouse PAR1-4 protein sequences showing the activating peptide, cleavage sites (residues variable between mouse and human receptors are shown in bold), and N-terminal affinity sites showing HLDs (red) for PAR1, PAR3, and the anion retention domain (gold) for PAR4. Also shown are protein sequences of the ECL2 domain; residues shared by four (green), three (blue), and two (orange) PARs are highlighted accordingly.

Protease-activated receptors (PARs) act as cellular sensors for the proteolytic state of the extracellular environment. The PAR family of G protein-coupled receptors (GPCRs) is comprised of four variants in mammals (PAR1-4) (Figures 1A–D), which can convert an extracellular cleavage event from a variety of proteases (12) into a transmembrane signaling event. This is mechanistically accomplished by the receptor carrying its internal ligand [i.e., tethered ligand (TL)] on its N-terminus (13). The ligand is masked by an exodomain, which prevents the receptor from signaling, with a protease cleavage site bridging the two domains (Figures 1E–H). Upon proteolytic cleavage, the exodomain is released, and the receptor signals through the interaction of the TL with the body of the receptor, specifically the second extracellular loop (ECL2) domain of the receptor (Figure 1H) (14). Dysregulation of PARs has been linked to numerous pathological conditions, including cancer, inflammation, and thrombosis (15).

PAR1 (Figure 1A), PAR3 (Figure 1C), and PAR4 (Figure 1D) can be activated by thrombin, whereas PAR2 (Figure 1B) is primarily activated by trypsin (15, 16). PAR3 is a co-receptor (17) and has a limited capacity to signal by itself (14), but it can enhance PAR1 and PAR4 signaling (17). PAR1 and PAR3 possess a hirudin-like-domain (HLD) on their N-termini (18, 19) (Figures 1E–F) and are the high-affinity receptors for thrombin (EC_50_: 50 pM and EC_50_: 200 pM, respectively) (16, 20). PAR4, on the other hand, is the low-affinity receptor for thrombin (EC_50_: 5,000 pM) (16) (Figure 1). The HLD mimics hirudin, a protein produced by leeches that acts as an anticoagulant (21); it binds exosite I of thrombin, which is distinct from the enzyme's active site that cleaves substrate peptides. Although PAR4 lacks an HLD, it does contain an anionic retention cluster (human: D_57_, D_59_, E_62_, D_65_, mice: D_69_, D_71_, E_74_) (Figure 1G), which slows the dissociation of cationic thrombin and prolongs the interaction time of thrombin with the receptor (22), allowing for bound thrombin to cleave the receptor more efficiently to initiate PAR4 signaling.

PAR1 is well characterized on ECs as the predominant mediator of thrombin signaling. PAR4, on the other hand, has minimal characterization in ECs, and there is significant controversy on whether it is even expressed in these cells (6, 23, 24). This review outlines what is known about PAR4 on ECs and provides a vision of how this controversial receptor signals in the endothelium based on recent breakthroughs in endothelial PAR4 research.

## A brief history of thrombin signaling

The ancient Greek medical philosophers Galen, Hippocrates, Plato, and Aristotle all attempted to interpret the phenomena of blood clotting and tried to understand its functional consequences (25). One of the earliest references to the word *thrombus* is found in Hippocrates's *Corpus Hippocraticum*, which refers to the “lump” rising from the coagulation of bodily fluids (25). By 1872, the German physiologist Alexander Schmit hypothesized the existence of an enzyme that converts fibrinogen into fibrin (26). At the time Schmit referred to the enzyme as *fibrin ferment* (27). By 1894, inactive prothrombin was isolated from plasma by the Dutch physiologist Cornelis Pekelharing (28). By 1954, platelets were shown to be activated by thrombin, demonstrating that the enzyme could not only mediate proteolytic cleavage but also cellular effects (29). Following the successful culture of ECs *in vitro* in the early 1970s (30), thrombin was also shown to have cellular effects on ECs (31).

However, it was not until 1991 that the thrombin receptor (later renamed PAR1) was identified (20). Yet, almost immediately, a limitation of the receptor was noted: given that PAR1 is quickly and irreversibly activated at extremely low concentrations of thrombin (EC_50_: 50 pM), how can it modulate variable responses to different thrombin concentrations (32)? Theoretically, PAR1 should always signal with zero-order kinetics at physiologic concentrations of thrombin, which ranges from 1 nM (0.1 U/ml) to 500 nM (50 U/ml) during coagulation (27). Furthermore, given that the majority of PAR1 molecules are rapidly removed from the cell surface following the receptor's activation (33), it is unlikely that persistent thrombin concentrations would have variable signaling responses. In 1993, Ishii and colleagues postulated that “*quantums*” of second messenger were produced following PAR1 activation, and cells may be able to detect balances between different rates of receptor activation and second messenger clearance, thus allowing for variable thrombin responses. However, a simpler answer was determined in 1998, with the identification of the low-affinity thrombin receptor, PAR4 (34, 35). With the identification of PAR4, it was then understood how cells could mediate responsiveness to high and low concentrations of thrombin using a system of dual receptors (36, 37).

## Controversy surrounding endothelial PAR4

By the early 2000s, PAR4 was shown to be present in murine pulmonary ECs, with PAR1 and PAR4 serving partially redundant roles in mediating thrombin responses in these cells (24). However, since 2003 there has been a paucity of studies identifying roles for endothelial PAR4. A PubMed search for “Endothelial”, “Protease”, and “PAR4” yields only 88 publications in the last 21 years; by comparison, the same period yields 615 publications related to endothelial PAR1. Of those 88 PAR4 publications, 16 claim they could not find a function for endothelial PAR4 in their respective models (38–53). This is likely due to a lack of suitable *in vitro* models since PAR4 shows limited expression and responses in human umbilical vein EC (HUVECs) (23), which are frequently used for *in vitro* EC studies. Additionally, endothelial PAR4 studies have been difficult to reproduce between labs (6, 54). For example, Vogel and colleagues found that a thrombin-induced increase in endothelial permeability was fully abrogated in *Par1*-deficient mice (54), unlike a similar study by Kataoka and colleagues that found partial redundancy between both PAR1 and PAR4 on ECs (24). The combination of low expression on ECs and mixed results regarding functionality has relegated PAR4 to be viewed as an irrelevant receptor on the endothelium—until now.

## PAR4 is a potent and functional receptor in the hepatic endothelium

We have recently shown that murine liver ECs express functional PAR4, albeit at low levels, with the *Par1:Par4* expression ratio in hepatic ECs being 153:1 (1). In a model of acetaminophen (APAP) overdose, the hepatic vasculature becomes compromised, which presents as increased permeability and erythrocyte congestion in the liver (1). Simultaneously, there is a rise in thrombin generation (1), which can lead to endothelial PAR activation. Using mice with conditional deletions for *Par1* and/or *Par4* in ECs, we showed that both receptors contribute independently to APAP-induced bleeding and permeability and that endothelial PAR1 and PAR4 act synergistically to drive APAP-induced permeability in the liver (1). Most importantly, we found that the loss of PAR4 in ECs was comparable to the loss of PAR1 in terms of phenocopying vascular protection against APAP-induced vascular dysfunction. Thus, even though *Par4* constitutes <1% of *Par* transcripts in hepatic ECs, it mediates a response equivalent to the other 99%, suggesting that endothelial PAR4 is not only functional but is extremely potent compared to endothelial PAR1. This may be due to PAR4 potentially being able to generate massive second messenger levels due to its lack of receptor desensitization following activation (55–57). Altogether, our studies show PAR4 to be a low-expressing but potent receptor on hepatic ECs (1, 58).

## Organotypic endothelial heterogeneity of PARs

### Organotypic expression of endothelial PARs

**Figure 2:**
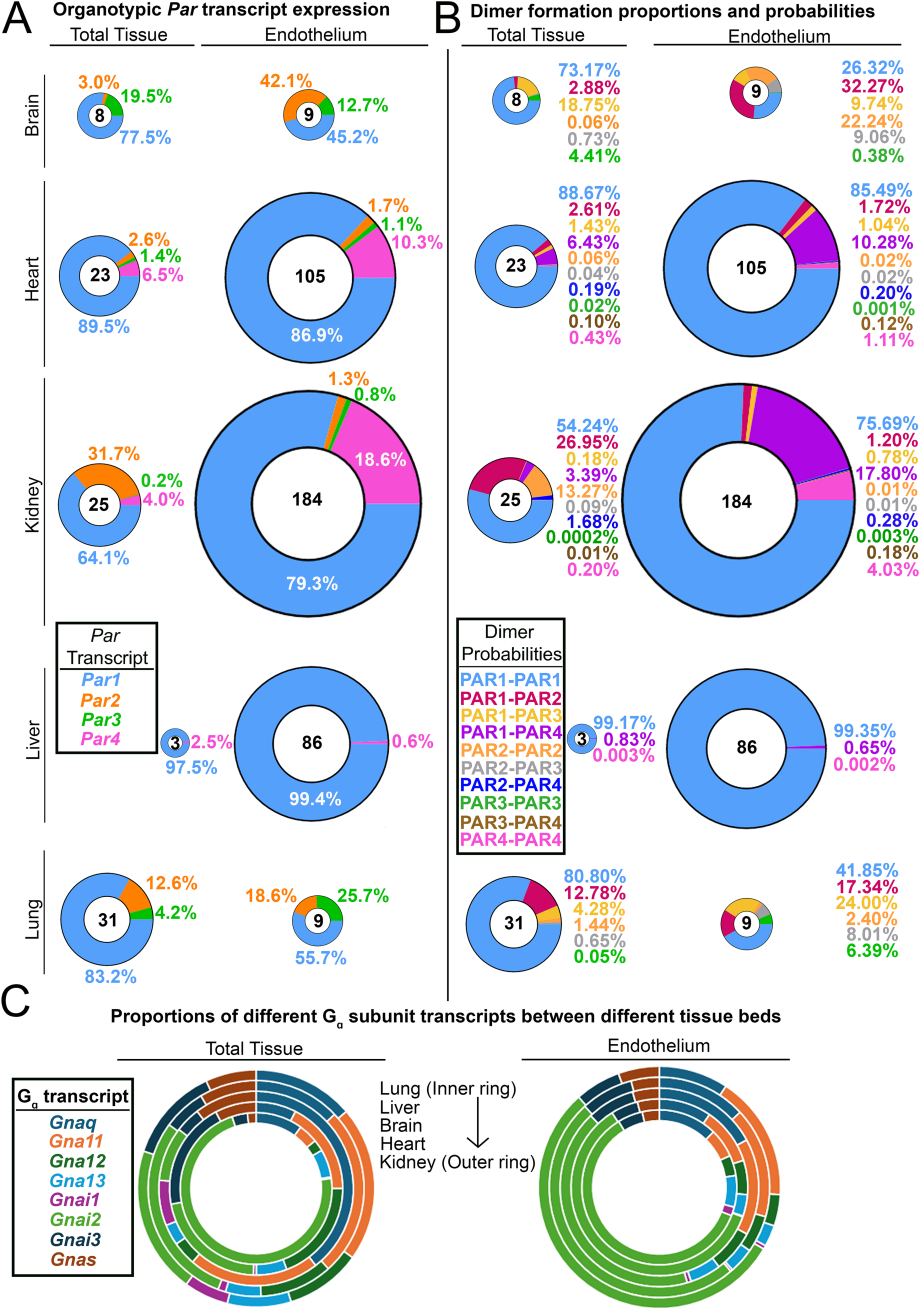
Organotypic differences in endothelial PARs and their G proteins. **(A)** Donut charts of transcript expression of all PARs in total tissues from different organs and the endothelium isolated from those organs. **(B)** Donut charts of PAR dimer formation probabilities in total tissues and endothelium from different organs. For **A** and **B**, the total transcript count for all PARs is shown in the center of each donut, and all donuts are sized to scale. **(C)** The proportion of G protein alpha subunit transcripts found in different organs and the endothelium isolated from those organs. Data for all organs in **A–C** (except for the liver) were generated by Cleuren et al. (59); liver data were generated by Rajala et al. (1).

ECs show remarkable heterogeneity in structure and function between different tissue beds (59). This also extends to organotypic gene expression. Given that PAR4 is functional at extremely low levels, an obvious question is whether these levels vary between ECs in an organotypic fashion. There were indications of this only a few years following the discovery of PAR4. In 2004, Fujiwara and colleagues reported the presence of PAR4 on aortic ECs but not on pulmonary artery ECs and HUVECs (60). However, there has never been a comprehensive study on this topic until our recent report (1). Using translating ribosome affinity purification (TRAP) datasets, we showed that *Par4* is expressed at low levels in ECs of most murine tissue beds *in vivo*, with transcripts per million mapped reads (TPM) values being substantially lower than *Par1* (1, 59)*.* Furthermore, endothelial *Par4* has organotypic differences in expression, with transcripts being limited to only a few of the organs that we analyzed in mice (Figure 2), unlike *Par1,* which is expressed ubiquitously among ECs of different organs (1, 59).

What appears to be more interesting is the organs in which PAR4 expression is undetectable *in vivo*, such as the brain and the lung. These are two organs in which alterations in permeability lead to severe injury via stroke or pulmonary edema, respectively. Given that PAR4 is highly potent, lung and brain ECs may rely only on PAR1 for thrombin signaling, as signaling with impunity (as PAR4 does) could be incompatible with preserving barrier function in these organs.

### Organotypic heterodimerization of endothelial PARs

All PARs have been shown to homo- and heterodimerize (61–63). This dimerization requires allosteric changes induced via thrombin-mediated receptor cleavage (61). However, the functional relevance of this dimerization is still an open question. Potential effects may include increased efficiency in activating protease recruitment (37), modified G-protein activity/second messenger production, altered dimerization-mediated coupling to different G-proteins (64), and differential internalization and trafficking of these receptors. Given the many possible variations in PAR dimer formations, careful combinatorial approaches to designing PAR experiments should take precedence over the assumption that specific dimers are responsible for cellular events.

As mentioned above, endothelial PAR expression ratios vary organotypically *in vivo* (Figure 2A). Assuming that homo- and heterodimerization kinetics are equivalent between all four receptors, one can easily determine the predicted formation probability of particular dimer products in organ-specific ECs based solely on expression ratios (Figure 2B). PAR1 homodimers are the most likely to form, with PAR1-PAR4 heterodimers being the second most likely in ECs of the kidney (∼18%) and heart (∼10%). In fact, the *Par1*:*Par4* expression ratio on renal ECs is 4:1, which is higher than the 5:1 PAR1:PAR4 receptor ratio on human platelets [PAR1; 2,500 copies/platelet (65): PAR4; 500 copies/platelet (66)]. Since the likelihood of PAR1:PAR4 heterodimer formation is high in renal ECs, this tissue may serve as a model system to study how PAR1/4 heterodimers influence EC function and signaling. Altogether, these results highlight the fact that since dimer formation has different probabilities in different tissues, identifying the function of endothelial PAR dimers may need to be done in an organ-specific manner.

### Organotypic expression of endothelial G proteins

One final category of organotypic variation that may affect endothelial PAR signaling is that of G protein alpha (G*_α_*) subunits. It has been hypothesized that the complexity that arises from GPCR signaling is due in part to variations in G*_α_* subunits (67). Theoretically, all receptors, when stimulated with the same ligand, should elicit similar responses. However, variations in how GPCRs couple to G proteins can result in diverse functional responses to the same ligand. We were curious if G*_α_* subunits—like PARs—varied between ECs of different tissue beds; if so, this could contribute to organotypic PAR responses. Interestingly, we found that the expression of different G*_α_* subunits does not vary in ECs of different organs (Figure 2C), although G*_α_* subunit expression does vary when compared across total tissues of different organs (Figure 2C). This suggests that if ECs display diversity in PAR responses, it may be driven more by organotypic variations in PAR expression as opposed to variations in the expression of the G*_α_* transducers to which PARs couple.

## Regulation of PAR4 expression in endothelial cells

Given that PAR4 is expressed at low levels but is functional in hepatic ECs, an obvious question is how its expression is regulated in ECs? Answers to this question may reveal reasons why PAR4 functions the way it does.

### Shear stress

We recently reported that *PAR4* expression increases around 13-fold in HUVECs exposed to shear stress and that this upregulation is comparable to that seen with the known shear stress responsive endothelial genes (Krüppel-like factor 2) *KLF2* and *KLF4* (1). Likewise, *PAR4* expression is increased *in vivo* in regions of high flow in the liver (1), as well as in vessels of high caliber (high flow) in the lung (68). If shear is a prerequisite for PAR4 expression, this may explain why static culture models have consistently failed to reveal the expression of PAR4 in ECs *in vitro* (23, 60).

**Figure 3:**
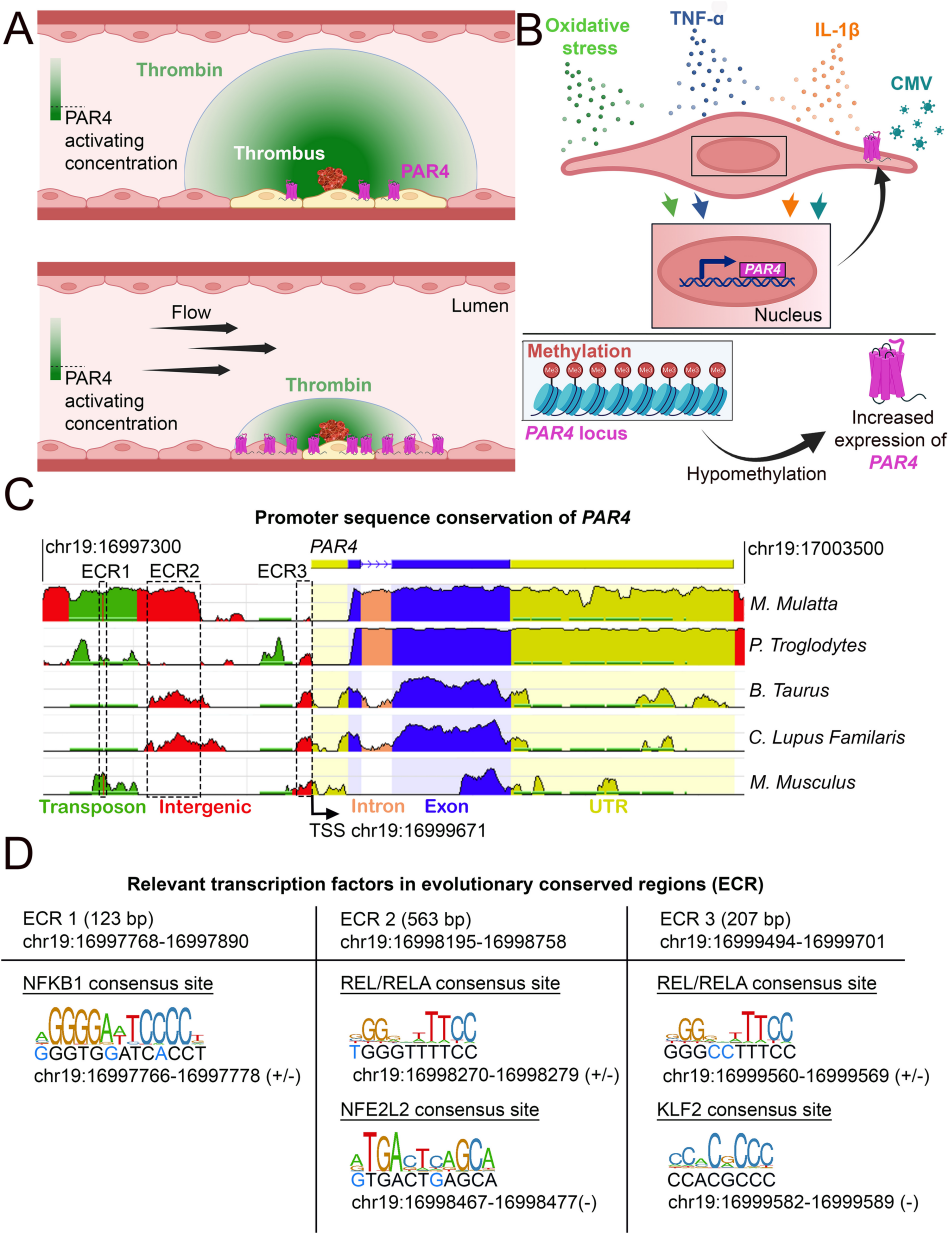
Conditions under which *PAR4* expression is altered. **(A)** Schematic of how endothelial *PAR4* (pink) increases with shear stress but how shear stress (flow) would limit the number of ECs activated by a point source of thrombin. The thrombin gradient is depicted in green. The use of differential color in ECs (yellow) depicts ECs engaging in PAR4 signaling. **(B)** Conditions under which *PAR4* expression has been shown to increase in ECs. **(C)** Analysis of evolutionarily conserved regions (ECRs) in the promoter of *PAR4* (human) between different species. Sequence homology was assessed using the NCBI DCODE website (http://www.dcode.org). Peak heights indicate the degree of sequence homology between species. Transposons (green), intergenic regions (red), intron (salmon), exons (blue), untranslated regions (yellow), and ECRs are annotated with dashed lines. **(D)** Relevant transcription factor binding sites (TFBS) in the three ECRs of *PAR4*. The sizes and locations of each ECR are given. Locations and strand(s) of each TFBS in the human genome are provided underneath each motif diagram. Nucleotides in the human genome that diverge from the motif sequence are identified with blue text.

This shear-induced upregulation of endothelial *PAR4* should be considered in the context of thrombin availability, which is diluted by blood flow. In an injured blood vessel, thrombin is generated from the cleavage of the zymogen prothrombin by FV or FX (69). This activation occurs locally near the injury site and does not occur systemically in circulation, as that could lead to disseminated coagulation. Additionally, thrombin is only active for less than one minute following prothrombin conversion (70) due to rapid inhibition by antithrombin in circulation (27). If high flow is maintained around the site of thrombin activation, it further decreases the local residency time of thrombin. Given that the activating protease residence time is proportional to the probability of thrombin encountering lumenal endothelial PAR4, the *constant dilution of locally generated thrombin* would decrease the likelihood of PAR4 being activated. Thus, under high shear stress—when ECs are likely to increase expression of PAR4—the likelihood of PAR4 being activated is decreased unless local thrombin concentrations are high enough to overcome dilution by blood flow (Figure 3A). Otherwise, under slow flow conditions, the low expression of PAR4 would help limit its activation.

### Inflammation and oxidative stress

Another major driver of *PAR4* expression in ECs is inflammation (Figure 3B). Cytomegalovirus-infected HUVECs demonstrate increased *PAR4* expression (71). Studies have also shown that both human coronary arteries and HUVECs treated with the inflammatory cytokines IL-1β or TNF-α have increased *PAR4* expression (72–75). In models of diabetic inflammation, increased vascular expression of PAR4 occurs (76–79). Similar effects can be seen in mice on a high-fat diet; elevated cardiac PAR4 expression correlates with increased IL-1β levels (80). Interestingly, PAR4 activation in cardiomyocytes results in increased TNF-α and IL-1β production, suggesting potential feedback between expression of PAR4 and inflammatory cytokines (81). However, this feedback mechanism has not yet been shown in ECs.

Cardiac microvascular ECs treated with homocysteine, which causes oxidative stress, show increased *Par4* expression (82). ECs isolated from patients with cerebral cavernous malformations, which originate from an environment of both inflammation and oxidative stress, also show increased *PAR4* expression (83). Thus, cytokine-mediated inflammation and/or oxidative stress are associated with increased PAR4 expression. One open question, however, is whether PAR4 signals the same under conditions of inflammation or shear stress. Future studies should focus on addressing this question.

### Epigenetic regulation

Cigarette smoking leads to DNA hypomethylation at the *PAR4* locus; this reduction in methylation is associated with increases in gene and protein expression for PAR4 (Figure 3B) (84). This hypomethylation has also been associated with an increased risk of death due to myocardial infarction (84). Furthermore, platelets from individuals with hypomethylation of the *PAR4* locus show increased reactivity to a PAR4 agonist (84). Independently, it has been shown that *PAR4* hypomethylation in DNA from blood cells is a strong predictor of all-cause mortality (85). Similar effects can be observed in cancer cells, in which *PAR4* is expressed only when the promoter is hypomethylated (86–88).

It is also noteworthy that *PAR4* is located on a different chromosome from the other PAR genes. *PAR1*, *PAR2*, and *PAR3* all map to Ch.5q13.3 and Ch.13D2 in humans and mice, respectively, whereas *PAR4* maps to Ch.19p12 in humans and Ch.8B3.3 in mice (89). Given the known methylation of the PAR4 genomic locus, it is possible that *PAR4*'s spatial separation from the other *PAR* genes further facilitates distinct and tight epigenetic and transcription control over its expression. Altogether, we predict that methylation is another way of suppressing potent *PAR4* expression on ECs, and it would be interesting to determine whether shear stress, inflammation, and oxidative stress can reverse this silencing epigenetic mark in pathological contexts.

### *PAR4* promoter conservation

Our cross-species analysis of the human *PAR4* promoter region revealed three evolutionary conserved regions (ECRs) (Figure 3C). Within these regions, we identified transcription factor binding sites (TFBS) for transcription factors linked to inflammation (NFBK1, REL/RELA), flow (KLF2), and oxidative stress [Nuclear factor erythroid 2-related factor 2 (NFE2L2)] (Figure 3D). These findings are consistent with these stimulatory effects leading to increased *PAR4* expression, as described above, and may indicate a role for these transcription factors in *PAR4* regulation.

### Heterologous downregulation

Using EC-TRAP, we have recently shown in murine hepatic ECs *in vivo* that PAR1 activation decreases *Par4* mRNA levels and that PAR4 activation decreases *Par1* mRNA (1). This can be interpreted as a form of heterologous downregulation (90) between both receptors. If endothelial PAR1 activation negatively regulates endothelial *Par4* expression at concentrations of thrombin that are suboptimal for PAR4 activation, it could mean that PAR1 serves as an extracellular thrombin sensor on ECs to limit accidental PAR4 activation.

## PAR4 trafficking habits and structural motifs reveal it to be a minimalistic receptor

GPCRs are regulated not only during transcription but also during the trafficking of the receptors to and from the cell surface, which controls their availability for signaling. Unlike other PARs, PAR4 appears to lack nuanced mechanisms for trafficking. However, we believe this is by design. Given the receptor's low expression and high potency, it would be unnecessary for PAR4 to evolve nuanced trafficking for various conditions. Rather, the receptor appears to only have one major function: transducing thrombin signaling at high thrombin concentrations. As such, we refer to PAR4 as a minimalistic receptor—one that is stripped of most of the trafficking levers found on other PARs.

### PAR4 lacks many post-translational modification sites required for trafficking

**Figure 4:**
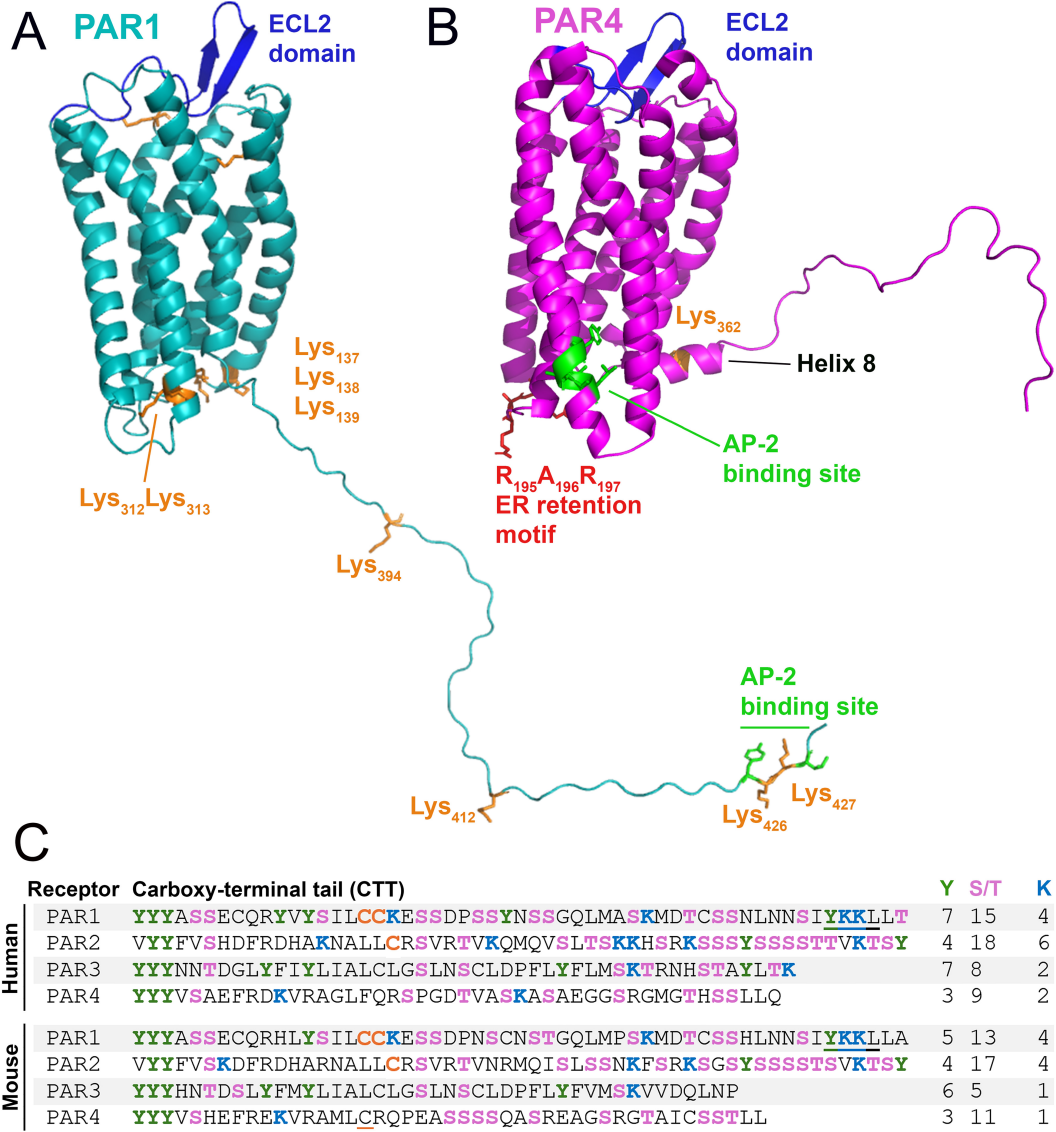
Structural elements of PAR1 and PAR4 that control trafficking. Major receptor trafficking elements of murine **(A)** PAR1 and **(B)** PAR4 are annotated. AP-2 binding site (green) and cytosolic lysines (orange), RXR retention motif (red). **(C)** Sequences of the C-terminal tail (CTT) of human and mouse PAR1-4: serine/threonine (pink); tyrosine (green); lysine (blue); palmitoylated cysteine (orange); putative palmitoylated cysteine is underlined in orange; AP-2 binding site (underlined). Numbers of tyrosine, serine/threonine, and lysine residues are listed at the right.

PAR4 is smaller in terms of residues (Human: 385; Mouse: 386) than PAR1 (Human: 425; Mouse: 430) and PAR2 (Human: 397; Mouse: 398). This indicates that PAR4 is a receptor that lacks many motifs which are present in PAR1 and PAR2. This size reduction is particularly noteworthy when observing the carboxy tail terminus (CTT) of PAR4, which is particularly short when compared to the CTTs of PAR1 and PAR2 (Figure 4). The CTTs of GPCRs often possess residues that engage with intercellular trafficking machinery (91).

#### Phosphorylation

Phosphorylation of GPCR CTTs by G protein-coupled receptor kinases (GRKs) is required for β-arrestin (βarr) recruitment, which aids in receptor desensitization and internalization. Notably, PAR4 contains fewer Ser/Thr sites on its CTT (Human: 9; Mouse: 11) compared to PAR1 (Human: 15; Mouse: 13) and PAR2 (Human: 18; Mouse: 17). PAR2 has the highest quantity of Ser/Thr sites and unsurprisingly has a greater dependency placed on βarr and CTT phosphorylation for its internalization (92, 93). The lack of phosphorylation sites on the CTT of PAR4 suggests that phosphorylation is not a major modality of internalization the receptor employs.

#### Ubiquitination

PAR1 and PAR2 trafficking is regulated by ubiquitination (94, 95). Human PAR1 has 10 cytosolic lysines (murine PAR1 has 9 cytosolic lysines) (Figure 4: Orange), and PAR2 has 14 cytosolic lysines (96). Ubiquitination of these lysines can alter internalization and degradation; in the case of PAR1, ubiquitination prevents degradation, while it promotes degradation of PAR2 (96, 97). However, PAR4 has very few lysines present on the receptor's CTT. *In silico* models of murine PAR4 (1) show only a single lysine (K_362_) to be present on the Helix 8 domain (H8) on the CTT (Figure 4). There are only two lysines on the CTT of human PAR4: one at K_350_, which matches the single lysine present on the mouse receptor on H8, and a second lysine at K_367_ (Figure 4C). This paucity of lysines on PAR4 may suggest the receptor has internalization dynamics that are not robustly affected by ubiquitination.

#### Palmitoylation

Palmitoylation is the addition of a palmitic acid moiety predominantly to a cysteine (or occasionally serine/threonine) residue via a thioester linkage. Palmitoylation of the CTT affects receptor trafficking in PAR1 and PAR2 (98–100). The palmitoylation of PAR1 occurs on dual cysteines (99) (Human: Cys_387_Cys_388_; Mouse: Cys_392_Cys_393_) in the CTT (Figure 4C). PAR2 also undergoes palmitoylation of a cysteine (Human: Cys_361_; Mouse: Cys_363_) on its CTT (100) (Figure 4C). Palmitoylation of both receptors has been shown to stabilize their expression on the cell surface (99, 100). It should be noted that human PAR4 lacks any cysteine residues on the CTT and that mouse PAR4 possesses only two cysteine residues (Figure 4C). Given that palmitoylation increases receptor stability at the cell surface, and that cells like ECs may seek to limit the expression of PAR4 except in circumstances of high thrombin levels, limiting opportunities for PAR4 palmitoylation may reduce the likelihood of its unintentional activation.

### AP-2 internalization

Following activation, PAR1 is rapidly internalized at a rate that is equivalent to or shorter than the time required for desensitization of its function (33). Although activated PAR1 is desensitized by βarr, it is not internalized in a βarr-dependent manner but in a distinct phosphorylation-, dynamin-, and clathrin-dependent pathway (101). This internalization is dependent on the clathrin adaptor protein complex-2 (AP-2) (102, 103). Following internalization, PAR1 rapidly passes through early and late endosomes before it is degraded in lysosomes (33, 104). The AP-2 binding domain of PAR1 (Human: Y_420_KKL_423_; Mouse: Y_425_KKL_428_) is located on the extreme C-terminus of the receptor (Figure 4) and binds the μ2 subunit of AP-2 (103).

Like PAR1, the internalization of PAR4 is not dependent on βarr but is dependent on AP-2 (105). However, unlike PAR1, in which the AP-2 binding site is found in the CTT, the AP-2 binding domain for PAR4 (Human: Y_264_GATL_268_; Mouse: Y_276_GATL_280_) is found in the intracellular loop 3 (ICL3) (105) (Figure 4). The fixed position of the ICL3 as opposed to the CTT, which has a free range of motion, would likely provide less AP-2 accessibility to its cognate binding site on PAR4 compared to PAR1. This limitation in AP-2 binding site accessibility may explain why PAR4 internalization and degradation have been observed to be slower than PAR1 (33, 105, 106). This in turn may explain why PAR4 has high potency on ECs and other cells, as the signaling half-life of the receptor on the cell surface is extended due to delayed internalization.

### PAR4 lacks N-terminal proteolytic termination sequences

PAR1 and PAR2 each contain numerous proteolytic cleavage sites on the distal end of their N-terminus, downstream of the tethered ligand (TL) and proximal to the first transmembrane domain of the receptor (12). These sites—often cleaved by neutrophil elastase, proteinase 3, and cathepsin G—are deactivation sites for these receptors (12), and upon cleavage, the TL is released from the receptor. Although the respective EC_50_ for thrombin and trypsin is high for PAR1 and PAR2 (i.e., pM-nM range), the equivalent EC_50_ for their TLs is relatively low (i.e., µM range). However, since the TL is physically attached to the receptor it triggers maximal signaling despite this low binding affinity. Using previously published models (1), we calculated the effective concentration for the TL of PAR1 to range from ∼71.3 mM - 39.4 M. However, proteolytic cleavage of the TL dissociates it from the receptor and leads to rapid receptor deactivation (15). It is noteworthy that PAR4 lacks these deactivation sites on its distal N-terminus, unlike PAR1 and PAR2. This lack of PAR4 deactivation sites suggests that the receptor was not designed for rapid signal termination and further explains its potency.

### Does PAR4 have an internal pool in ECs?

PAR1 is internalized both constitutively and upon activation. The constitutive internalization (i.e., tonic cycling) of PAR1 is critical for the generation of an internal pool of uncleaved receptors (107), which allows for the rapid replenishment of fresh receptors on the cell surface after proteolytic cleavage (33, 108). In ECs, this internal pool of PAR1 is ten times larger than the cell surface pool of the receptor (109). However, there is limited information on whether PAR4 has an internal pool. Given the receptor's high potency on ECs, we speculate that an internal pool of PAR4 may be unnecessary since there may be a lesser need for rapid replenishment of PAR4 on the cell surface following a PAR4 signaling event. Cells may choose to space out their PAR4 signaling events due to the receptor's potency. Furthermore, given the low expression of *Par4* transcripts in ECs, even if an internal pool existed, its small size may preclude our ability to detect it. Future studies focusing on whether ECs lack an internal pool of PAR4 would be beneficial for determining how the receptor is trafficked and whether cell surface repopulation requires *de novo* synthesis of the receptor.

### Is PAR4 a molecular hitchhiker?

PAR4 appears to take advantage of the movement of other receptors to traffic itself (110, 111). We term this behavior as *receptor phoresy.* Phoresy is defined as an interaction in which a phoretic animal (i.e., a phoront) latches itself onto a host for travel. The word is derived from the Greek *phorein*, meaning “to carry” (112). In the case of PARs, we argue that PAR4 is likely a phoront to other PARs. As a phoront, PAR4 would benefit from the regulation and trafficking of other receptors, without needing to carry specific trafficking motifs on itself. This behavior has been directly observed in the case of PAR2 and PAR4, with heterodimerization of these receptors resulting in more efficient PAR4 migration to the cell surface (110). In the absence of PAR2, PAR4 is retained in the ER via its arginine-based (RXR) ER retention motif in the second intracellular loop (ICL2) of the receptor (Figure 4). This suggests that PAR4 requires the assistance of a cofactor to migrate from the ER to the cell surface unless this domain is masked. However, in murine hepatic ECs—where PAR4 is known to be functional—we detect little to no expression of PAR2 (1) (Figure 2A). Thus, it may be an unlikely trafficking partner for PAR4 in these cells. Alternatively, PAR4 may be trafficked alongside PAR1 to the cell surface in hepatic ECs, particularly if stable heterodimers of PAR1 and PAR4 can form without thrombin-mediated cleavage, as seen in platelets (63).

Similarly, there is an open question as to whether heterodimerization of PARs alters their internalization. Overexpression studies have shown that PAR1 and PAR4 heterodimerize upon thrombin-induced cleavage (61). This allows the HLD of PAR1 to capture exosite I of thrombin and localize its active site to the exodomain of PAR4, effectively amplifying PAR4 signaling and decreasing its EC_50_ ∼2.9-fold (63). However, since proteolytic cleavage results in receptor internalization, it is not known whether the PAR1/4 heterodimer gets internalized as a complete unit or whether each monomer is internalized separately. In the former case, PAR4 may be internalized as a phoront alongside PAR1 with more rapid kinetics, thus limiting the signaling lifetime of PAR4. Future studies focusing on whether PAR4 is a phoretic receptor would be beneficial to determine how exactly this receptor is trafficked to and from the membrane.

## How does PAR4 signal in endothelial cells—is it redundant to PAR1?

### Different roads that lead to Rome

**Figure 5:**
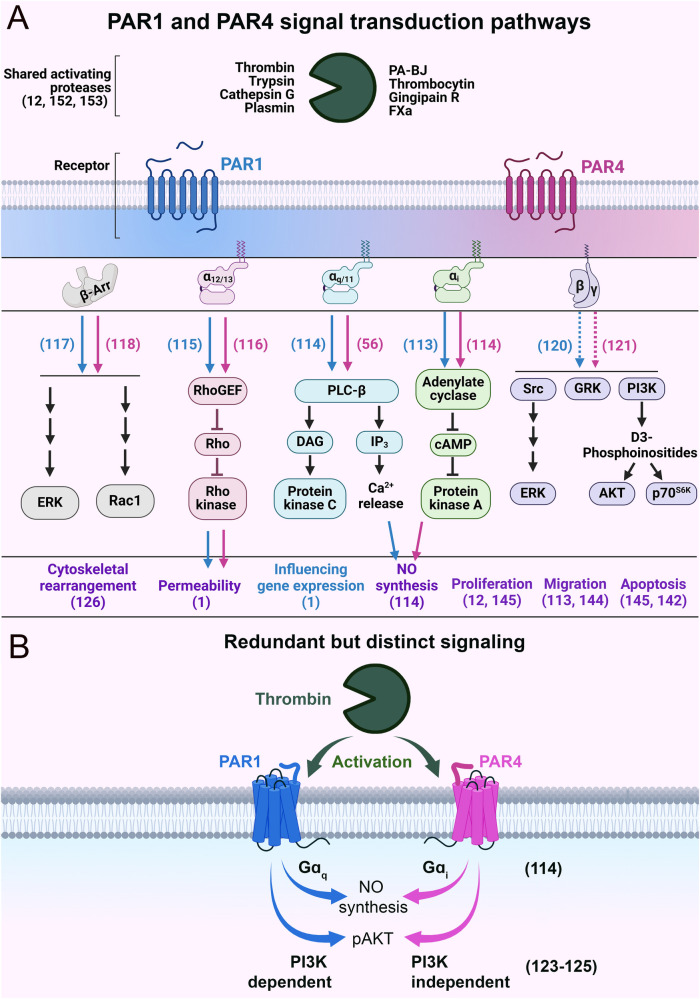
Schematics of PAR1 and PAR4 signal transduction pathways. **(A)** The major intracellular signaling events and biological effects following the activation of PAR1 and PAR4. Blue and pink arrows indicate PAR1- and PAR4-specific signaling, respectively. The purple text refers to the biological impacts mediated by PAR1 and PAR4. The blue text refers to biological effects mediated by PAR1 alone. Dashed lines indicate hypothesized behavior. **(B)** Schematic of functionally redundant but mechanistically distinct signaling of PAR1 and PAR4. Relevant citations are shown in parentheses.

PAR1 and PAR4 are promiscuous receptors with the ability to couple with multiple G protein subunits [G*α*_i_ (113, 114), Gα_q/11_ (56, 114), Gα_12/13_ (115, 116)], as well as βarr (117, 118) and likely G_βγ_ (119–121) (Figure 5A). Traditionally, PAR4 was seen as a providing redundant function to PAR1 (122); however, studies have shown nuanced differences in the signaling of these receptors. Notably, these receptors can utilize different mechanistic paths to reach the same cellular destination (i.e., functional outcome). For example, PAR1 and PAR4 can also mediate the same function using different G proteins (Figure 5B). In bovine arterial ECs, PAR4 uses Gα_i_/adenylyl cyclase signaling to mediate nitric oxide production, whereas PAR1 utilizes Gα_q_/calcium signaling for this function (114). Similarly, in platelets, PAR4 activation mediates AKT phosphorylation independent of phosphoinositide 3-kinase (PI3K) (123), whereas PAR1 mediates AKT phosphorylation through PI3K (124, 125). In cultured human pulmonary ECs, PAR4 regulates actin cytoskeletal rearrangement, which can also potentially regulate permeability (126). The mechanism for this cytoskeletal rearrangement is likely via Gα_q_/phospholipase C-β (PLC-β)/calmodulin/myosin light chain kinase (MLCK) (127) or Gα_q_/RhoA-GTPase/p38/ERK/MAPK (128) or Gα_12/13_/Rho-GTPases signaling, in a similar manner to PAR1 (129). However, PAR4 has also been shown to regulate cytoskeleton rearrangement independently of G proteins in a βarr/RhoA-dependent pathway (118) or through a PI3K/Rho-dependent pathway (130). Overall, we hypothesize that even though PAR4 can be redundant to PAR1, it can utilize alternative signaling pathways to achieve the same functional outcome.

These distinct mechanisms are reasonable given that PAR4 is a low-affinity receptor when compared to PAR1 (100-fold). Teleologically, a scenario can be imagined in which thrombin concentrations are high enough for PAR4 to be activated, but such concentrations would also result in nearly all the PAR1 on the cell surface getting cleaved and activated. In such a scenario, having PAR4 compete for second messenger resources with PAR1 would be counterproductive for signal transduction. Therefore, allowing for alternate pathways/G-proteins to transduce signal would allow PAR4 to signal efficiently even when PAR1 signaling is saturated. This is akin to a higher gear setting in a vehicle. As switching from a low gear to a high gear allows for more efficient transmission of power from the engine to the wheels, switching from PAR1 to PAR4 signaling allows for more efficient transduction of thrombin signaling to an EC.

### PAR4 has limited roles in influencing endothelial gene expression

Recently we showed that in hepatic ECs, PAR1 can influence gene expression following activation, but PAR4 cannot. We found PAR1 activation resulted in upregulation in transcripts linked to permeability and cytoskeletal rearrangement, while PAR4 activation did not alter transcription in ECs (1). This is reasonable given that PAR4, unlike PAR1, is a potent receptor with limited desensitization (55). If PAR4 could influence gene expression, its persistent signaling could result in significant fluctuations in gene expression (56). Given that ECs can survive for years *in vivo* (131) and may encounter multiple thrombin signaling events during their lifetime, PAR4-mediated persistent transcriptional events in these cells could be deleterious to their long-term function and homeostasis.

## Open questions in endothelial PAR4 biology

### Is endothelial PAR4 mechanosensitive?

**Figure 6:**
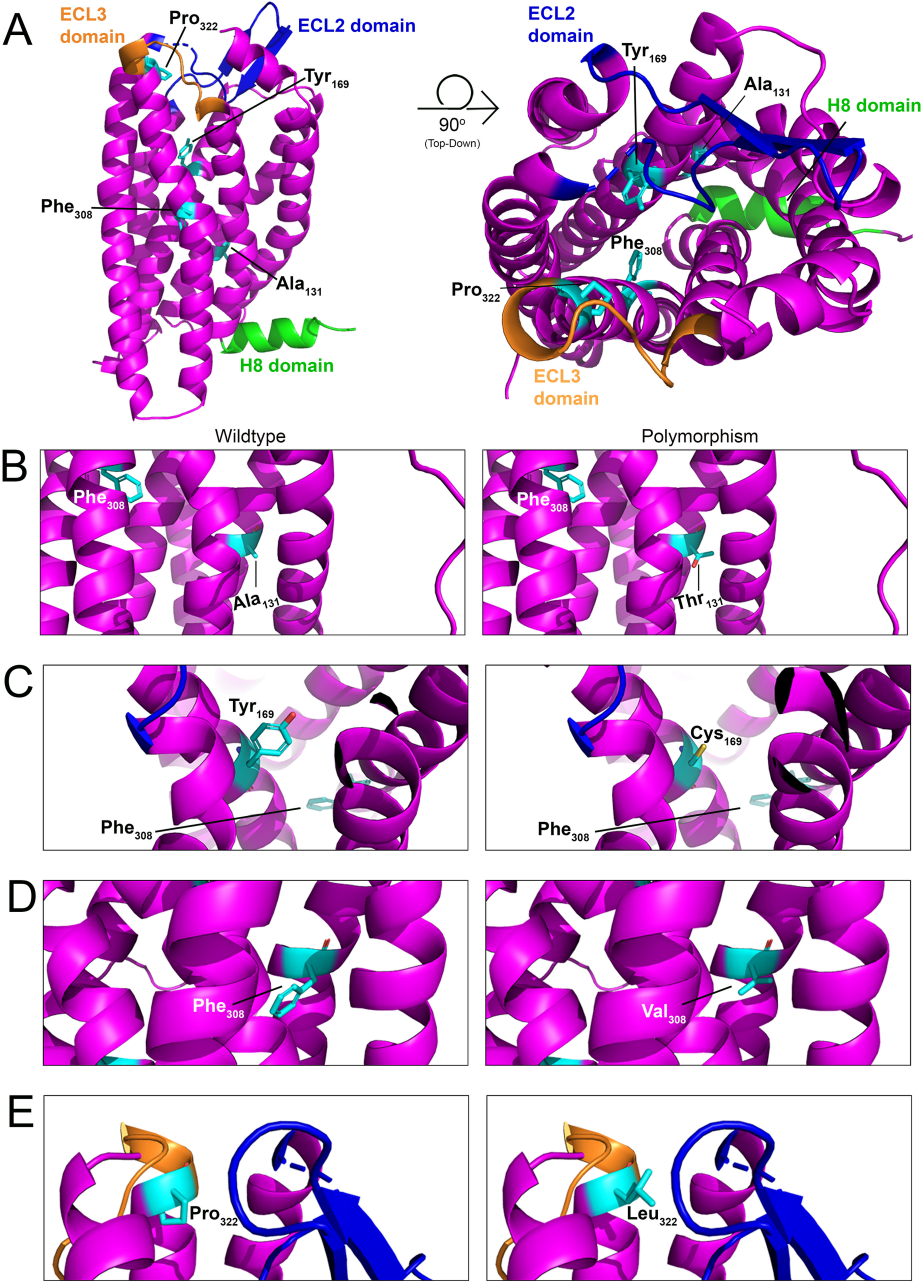
Structural model of PAR4 single nucleotide polymorphisms (SNPs). **(A)** *In silico* models with 90-degree rotation (top-down view; right) of murine PAR4 with common SNPs shown in cyan. The Helix 8 (H8) domain of the receptor is shown in green, the extracellular loop (ECL) 2 domain is shown in blue, and the ECL3 domain is shown in orange. Wildtype receptor (left) and polymorphism-carrying receptor (right) are depicted for **(B)** Ala_131_Thr, **(C)** Tyr_169_Cys, **(D)** Phe_308_Val, and **(E)** Pro_322_Leu.

GPCRs are defined as heptahelical proteins, but some have an eighth alpha helix (H8) in their CTT. This H8 domain can have mechanosensing properties. Although there is a lack of sequence conservation or defined length for the mechanotranducing H8, the domain is always parallel to the inner leaflet of the cell surface (132) and is critical for sensing mechanical stretch forces in cells. For example, the mechanosensitive histamine H1 receptor (H_1_R) is an endothelial sensor of fluid shear stress through its H8 domain (133). *In silico* models of murine PAR2, PAR4, and to a lesser extent PAR3 also show the presence of this domain (Figures 1A-D). Interestingly, the PAR4 H8 domain (Figure 6A: green) has a similar homology (Mouse: EFREKVRAML | Human: EFRDKVRAGL) to the mechanosensitive H_1_R GPCR (ENFRKTFKRIL), with 30% fully conserved residues and 70% conservation of amino acid groups of similar properties. However, there are no studies that focus on whether/how flow affects PAR4 signaling. As mentioned previously, *PAR4* expression is regulated by shear stress in HUVECs, but whether PAR4 signaling is altered during flow is still an open question. Given the fact that this highly potent receptor is expressed on ECs, which are constantly exposed to and respond robustly to variable flow rates, future studies focusing on how flow alters endothelial PAR4 signaling in different contexts would be beneficial.

### Do PAR4 SNPs affect endothelial cell function?

Four single nucleotide polymorphisms (SNPs) have been identified within PAR4 that result in its altered expression and function in platelets (17, 134). However, nothing is known about how these SNPs affect PAR4 in ECs. *Ala*_*120*_*Thr (rs773902*): This mutation is found on the second transmembrane of PAR4 and leads to its increased functional responses in platelets (135). The equivalent residue in mice is Ala_131_ (Figure 6B). *Tyr*_*157*_*Cys*: This mutation, which is found on the third transmembrane domain of the receptor, leads to an attenuation of PAR4 functional responses in platelets (135). The equivalent residue in mice is Tyr_169_ (Figure 6C). *Phe*_*296*_*Val (rs2227346)*: This mutation is less common and has only been observed in Black individuals (17, 136). The mutation is present on the sixth transmembrane domain and leads to decreased PAR4 second messenger production in response to receptor activation (135). The equivalent residue in mice is Phe_308_ (Figure 6D). *Pro*_*310*_*Leu (rs2227376)*: This mutation is found on the extracellular loop 3 of the receptor and leads to an attenuation of PAR4 functional responses in platelets (137, 138). The equivalent residue in mice is Pro_322_ (Figure 6E).

Most of these SNPs produce inactivating mutations. Given that endothelial PAR4 appears to signal as a thrombin relief receptor (i.e., it signals only when PAR1 is saturated), there may be sufficient PAR1 on ECs to mitigate the loss of PAR4 function. The more concerning mutant is the Ala_120_Thr, which is a hyperactive mutation that increases PAR4 activity. Given the high basal potency of PAR4 on ECs, hyperactivation may lead to ECs that are more sensitive to thrombin-mediated endothelial dysfunction (79). Future studies focusing on whether the Thr_120_ variant of PAR4 results in EC hyperreactivity to thrombin would be beneficial.

### Does PAR4 mediate apoptosis and regression in ECs?

Another interesting finding is that endothelial *PAR4* expression increases in response to inflammatory cytokines, such as IL-1β and TNF-α. Also of note is that the combination of IL-1β, TNF-α, and thrombin treatment robustly drives the regression of capillary tubes grown in a 3D culture model (139). In the case of lymphatics, TNF-α, IFN-γ, and thrombin also promote capillary tube regression (140). Given that proinflammatory cytokines selectively increase *PAR4* expression in ECs, it is possible that capillary regression may be driven less by thrombin activation of PAR1 than by thrombin activation of PAR4.

Similar effects are observed in cancer cells, in which PAR1 activation promotes tumor growth and metastasis, while PAR4 acts as a tumor suppressor that inhibits tumor growth and metastasis (141). This could be explained, in part, by the fact that PAR4 activation increases protein expression of apoptosis factors (e.g., caspase 9) (142). This apoptotic function of PAR4 is present in esophageal (142), gastric (87) and lung (143) cancers, although this effect is not universal for all cancers as colorectal cancers show increased proliferation with increased expression of PAR4 (144). Furthermore, other studies have shown that in certain cases PAR1 activation can also induce apoptosis, suggesting PARs can diversely influence cell death depending on varying conditions (145).

### Do endothelial PARs affect thrombosis and hemostasis?

**Figure 7:**
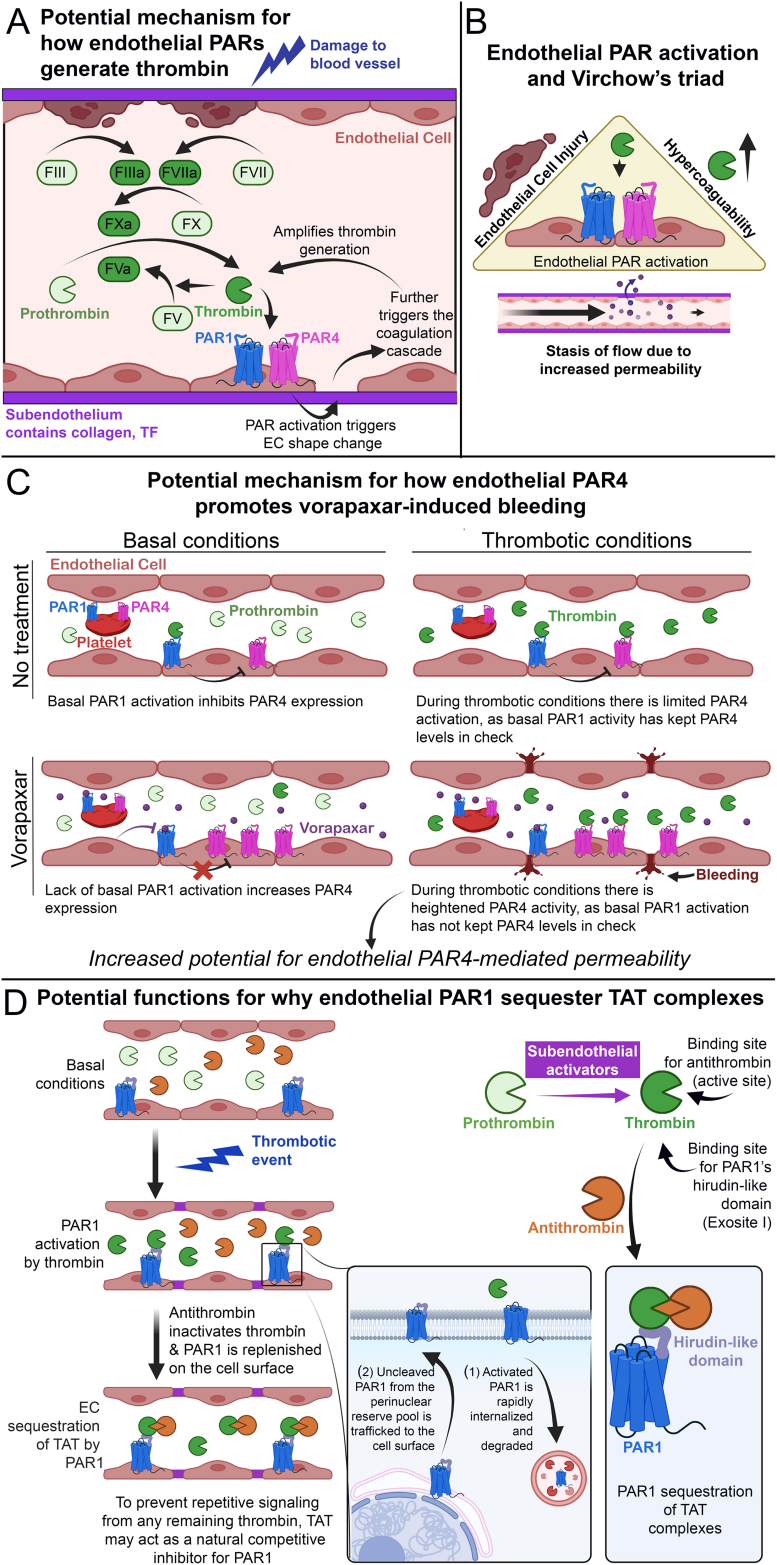
Endothelial PAR4 and thrombosis. **(A)** Schematic of how endothelial PARs potentially amplify thrombin generation after initial blood vessel damage. **(B)** Schematic of how endothelial PAR activation potentially increases thrombosis by promoting the factors of Virchow's triad. **(C)** Schematic of how endothelial PAR4 may potentially contribute to vorapaxar-induced bleeding in humans. **(D)** Schematic of how endothelial PAR1 may act as a molecular sink for TAT, subpanel adapted from Rajala et al. (1).

It is well-known that thrombin activates PAR1 and PAR4. However, what is less understood is the effect endothelial PARs have on thrombin generation and hypercoagulability in general. We have recently shown that in a model of APAP overdose (1)—which presents with increased thrombin levels and hypercoagulability, as measured by plasma thrombin-antithrombin (TAT) levels—that loss of both endothelial PAR1 and PAR4 reduces thrombin generation. However, mice lacking in endothelial PAR1 or PAR4 alone do not demonstrate reduced thrombin levels after APAP overdose, suggesting that both endothelial PAR1 and PAR4 work synergistically to promote thrombin generation. We speculate that endothelial PAR1/4-mediated thrombin generation results in cytoskeletal rearrangement of ECs (Figure 7A), resulting in exposure of subendothelial coagulation initiators such as collagen and tissue factor. This results in a feedback mechanism in which thrombin generation can be amplified by the actions of endothelial PAR1 and PAR4. In a separate study, we also found that PARs act synergistically between multiple cell types, including the endothelium, to promote hemostasis (146), further suggesting *bona fide* roles of endothelial PARs in mediating hemostatic responses.

This form of regulation can also directly impact thrombosis. The modern understanding of thrombosis can be observed in the lens of *Virchow's triad* (147), which outlines the three broad contributing factors: hypercoagulability, endothelial injury, and stasis of flow (Figure 7B). All three of these factors are promoted by the activation of endothelial PARs. *Hypercoagulability*: Our data from APAP-overdosed mice indicate that endothelial PAR4 can contribute to hypercoagulability *in vivo* (1). *EC injury*: Overpotentiating PARs has been shown to mediate pro-inflammatory signaling (128) that can lead to EC injury (76). *Stasis of flow*: It has been shown that alterations in permeability can also alter flow rates in ECs (5, 148, 149). This is most notably observed in tumor vasculature, in which leaky vessels demonstrate increased angiogenesis alongside impaired flow (150). Furthermore, our recent work demonstrates that endothelial PAR1 and PAR4 promote vascular permeability after APAP overdose (1), and a result of this permeability may be impaired vascular flow. Thus, endothelial PARs fit the bill as factors that promote thrombosis and may represent novel therapeutic targets for reducing thrombotic activity.

### Does endothelial PAR4 contribute to vorapaxar-induced bleeding?

Vorapaxar is an orthosteric PAR1 inhibitor that was designed as an antithrombotic drug because of PAR1 expression on human platelets (151). The principle of inhibition stemmed from PAR1 and PAR4 being dual thrombin receptors on human platelets (152, 153). By inhibiting high-affinity PAR1 with vorapaxar, accidental thrombosis triggered by low concentrations of thrombin can be prevented (122). Meanwhile, since low-affinity PAR4 is still functional, high concentrations of thrombin generated during injury can still allow for hemostasis via platelet PAR4.

However, the use of vorapaxar presents with risks of increased bleeding (154). Our recent article on APAP overdose demonstrated that ECs have low-expressing but functional PAR4 *in vivo*. These low levels are further reduced by PAR1 activation and heterologous desensitization (1). We argue this is a negative feedback loop between thrombin/PAR1 signaling and *Par4* expression to limit PAR4 activation. Given that PAR4 is a potent receptor, ECs are invested in limiting its activation. Thus, in the case of low thrombin levels, subsequent endothelial PAR1 activation may act as a *brake* on PAR4 activation. Furthermore, studies in oligodendrocyte progenitor cells and astrocytes indicate that PAR1 has basal activity independent of thrombin stimulation (155). This suggests that PAR1 may be a leaky receptor, and perhaps PAR1 basal activity may be sufficient to act as a brake on PAR4 activity. Regardless of the mode of PAR1 activation, since vorapaxar effectively inhibits global PAR1 activation [including in ECs (156)], this may release the brake on endothelial PAR4. This potential for increased PAR4 activity on ECs may contribute to the increased bleeding seen in patients treated with vorapaxar, as it would result in unmitigated PAR4-mediated vascular dysfunction (Figure 7C). Overall, these findings raise questions about the interplay between PAR1 and PAR4. Understanding how these receptors interact with each other will be necessary to design effective and safe PAR therapeutics.

### Can a TAT complex act as a PAR1 inhibitor?

In a recent study from our lab, we found evidence that endothelial PAR1 may act as a molecular “sink” to bind and sequester TAT complexes *in vivo* (1). TAT complexes are formed by the binding of antithrombin to thrombin's active site (157). In contrast, PAR1 binds to thrombin's exosite I via the receptor's HLD (19) (Figure 1E). Given that exosite I is still accessible in TAT complexes (157), we hypothesized that TAT could bind and be sequestered by endothelial PAR1 on the lumenal surfaces of blood vessels (1). Leger and colleagues have previously proposed functional sequestration of thrombin by PAR1 in a model of PAR1/4 dimerization (63). In this model, PAR1 cleavage and activation are followed by retention of thrombin via HLD-exosite I interactions. This retention allows for thrombin-mediated cleavage of neighboring PAR4 molecules and lowers the EC_50_ for PAR4 activation ∼2-fold (56, 63). Whether PAR1 similarly binds TAT as it does thrombin is unknown. However, we believe PAR1-TAT interactions could represent a novel mechanism for PAR1 inhibition (Figure 7D).

In the endothelium, following a thrombotic event, thrombin cleaves PAR1 and drives rapid internalization and degradation of the receptor (33). PAR1 is subsequently replaced on the cell surface with new and uncleaved PAR1 molecules from an intracellular pool (106). At the same time, circulating antithrombin binds and inactivates thrombin, forming TAT complexes. We propose that the newly formed TAT complexes might then bind the newly trafficked PAR1 on endothelial surfaces to inhibit further thrombin-mediated activation and prevent repeated endothelial PAR1 signaling in response to the same stimulus. Circulating TAT might also bind PAR1 on human platelets or PAR3—which also contains an HLD (Figure 1F)—on mouse platelets, potentially inhibiting thrombin-mediated platelet activation. This may explain why we observed that mice with elevated circulating TAT also displayed protection against thrombocytopenia following an acetaminophen-induced thrombotic challenge (1).

Currently, the only FDA-approved PAR1 inhibitor, vorapaxar, is no longer available in the U.S. due to significant adverse bleeding events (154). Issues with vorapaxar included its high binding affinity and long half-life in the body, which resulted in the drug acting as a functionally irreversible inhibitor (158) and likely contributed to its side effects. If TAT complexes act as natural and competitive inhibitors to PAR1, new compounds designed to mimic how TATs inhibit PAR1 may avoid some of the negative effects associated with vorapaxar. Such compounds—which we refer to as inactive thrombin mimetics (ITMs)—would be proteolytically inert and carry the exosite I motif of thrombin, thus allowing for reversible binding and inhibition of PAR1 while not interfering with endogenous thrombin activity.

## Discussion: PAR4 is a PARsimonious receptor on the endothelium

Over the last two decades, endothelial PAR4 has been an enigma. Although some studies have shown the presence of PAR4 on ECs, its lack of expression *in vitro* and a lack of sensitive tools to assess receptor function *in vivo* have hindered research in this field. In this review, we show that the low expression of PAR4 may be purposeful. PAR4 is a very potent receptor, and ECs appear to employ numerous mechanisms to prevent its unintended activation except in cases of exceptional thrombin activity. As a result, ECs appear to take a parsimonious approach when employing PAR4 signaling by limiting the expression of the receptor. This is in stark contrast to platelets, in which PAR4 is robustly expressed (159) and was first detected (34). This lack of limitation on platelet PAR4 expression is reasonable when you consider that thrombin-mediated activation of platelets *is a terminal event* (160); platelets cease functioning and irreversibly aggregate following PAR4 activation. Meanwhile, thrombin activation of ECs *is a temporary event;* ECs are functional for years (131) and likely experience multiple PAR4 signaling events during their lifetime. Furthermore, unlike platelets, ECs need to survive to maintain blood vessel integrity, and an extensive second messenger response could be harmful to endothelial function and survival. The dichotomy between the terminal and the temporary could explain why tight regulation and low expression for PAR4 exist in ECs but not in platelets. Understanding this dichotomy is fundamental to understanding how PAR4 functions on ECs. Thus, for endothelial PAR4, we argue its low expression is not indicative of a failure to function, but rather, it appears to be a feature of the receptor to ensure that PAR4 signaling does not have deleterious consequences.

**Figure 8:**
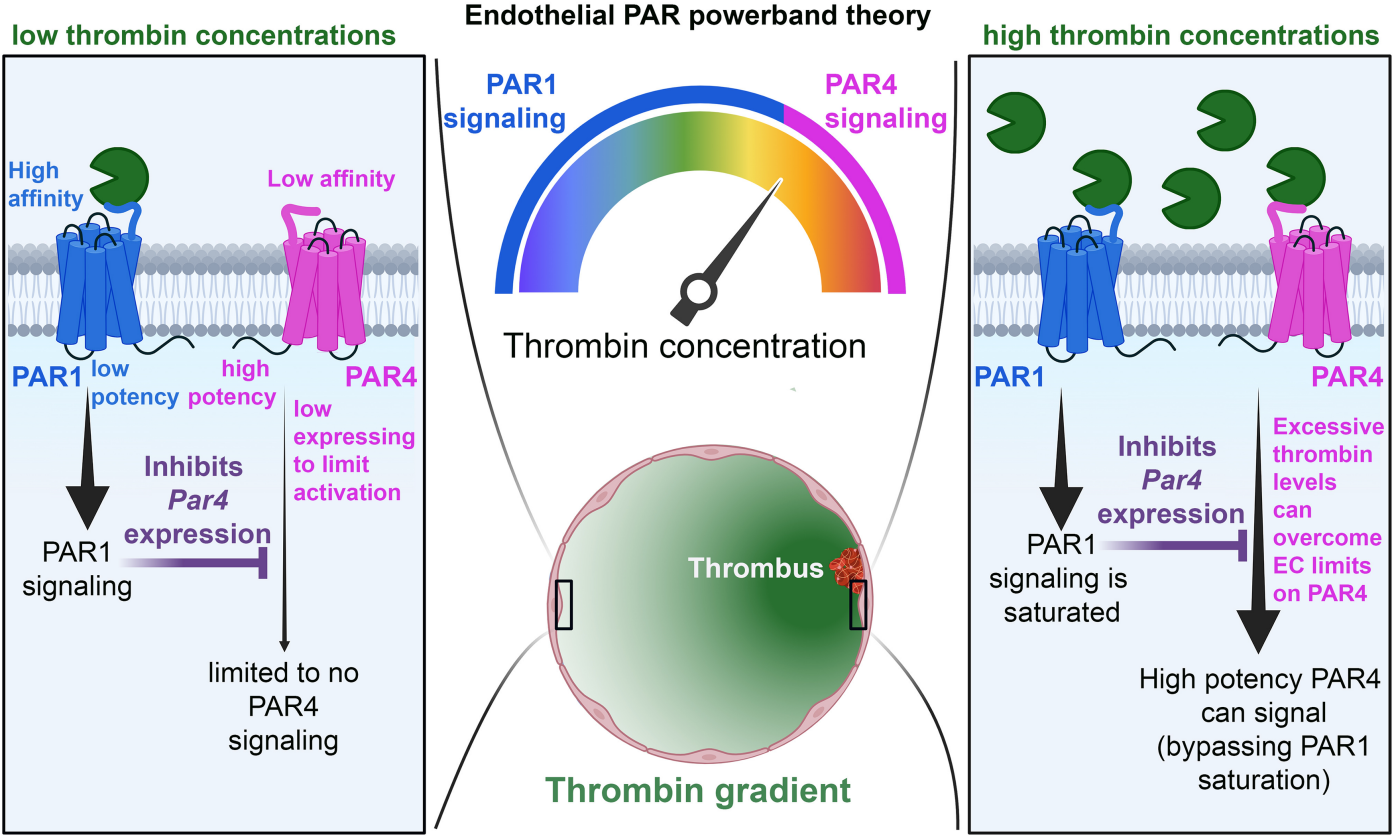
Schematic of the PAR powerband theory. In conditions of low thrombin activity (left), PAR1 is allowed to signal, and its activity inhibits PAR4 expression. This, coupled with low PAR4 expression and affinity for thrombin, prevents PAR4 from signaling on ECs. In conditions of high thrombin activity (right), PAR1 activation becomes saturated, and endothelial PAR4 is allowed to signal with high potency.

However, in cases when PAR4 is allowed to signal, it signals with impunity—using distinct signaling pathways from PAR1. This allows PAR4 to mediate continuous transduction of thrombin signaling, even in high thrombin environments, where PAR1 signaling is saturated (Figure 8). Therefore, the presence of PAR4 on ECs allows for access to higher *powerbands* of thrombin signaling. Nevertheless, this potency comes at a cost. PAR4 appears as a *minimalistic receptor*, in that the receptor does not appear to be designed to engage in nuanced post-translational regulation, unlike PAR1 and PAR2. There is a lack of cysteine, lysine, serine, and threonine residues on the CTT as well as a lack of lysines in the AP-2 binding site, suggesting a lesser role for palmitoylation, ubiquitination, and phosphorylation of the receptor. PAR4 may also be reliant on other PARs for trafficking. This may all reflect the likelihood that endothelial PAR4 is designed with a singular task in mind: transducing massive amounts of second messenger at high concentrations of thrombin. Thus, there is no need for PAR4 to demonstrate the nimbleness and elegance seen in PAR1 and PAR2 trafficking. In the case of PAR4, the cell needs only to place a low number of receptors on the cell surface and allow the receptor to signal with impunity. However, to compensate for the lack of posttranslational control placed on PAR4, ECs likely exercise significant transcriptional control on the receptor, which may explain why its levels are so low in ECs.

Taking all this into consideration, we argue that PAR4 is not an impotent receptor on the endothelium, but rather an important one whose regulation reflects its roles and functions.

## Future directions

### *In vivo* focus for endothelial PAR4 studies

Although mechanistic insights about PAR4 would theoretically be easier to explore *in vitro*, assigning roles for receptor function and expression based on cell culture models appears to result in spurious and contradictory results. Future studies should focus more on the receptor in an *in vivo* context with organotypic expression in mind, as this approach yields the most accuracy in assigning/identifying the cellular functions of the receptor.

### How does PAR4 affect the endothelial phosphoproteome/kinome?

Protein phosphorylation is a significant regulator of protein function in all cells; kinases, phosphatases, and relevant regulatory subunits constitute 2.5% of all human genes (161). Given that we have previously shown that endothelial PAR4 has an extremely limited ability to influence transcription (1), PAR4 likely only mediates downstream signaling effects through post-translational modifications such as phosphorylation. Currently, there have been a few studies that focus on the impact of PAR1 activation on protein phosphorylation (156, 162, 163). Notably, Lin and colleagues looked at a bias in the phosphoproteome between activated protein C (aPC) and thrombin-mediated PAR1 activation (162). Identifying phosphoproteomic signatures following endothelial PAR4 activation and comparing them to signatures from PAR1 activation would allow us to gain a deeper understanding of receptor-specific actions. This was attempted in a recent study by Groten and colleagues using cultured ECs and PAR1/4-specific agonist peptides, and the authors found limited PAR4-mediated effects on protein phosphorylation (163). However, this may be due to the lack of flow (i.e., shear stress) used in these studies, which we detailed above as being important for PAR4 expression on ECs. Future studies may need to employ shear to determine PAR4-specific phosphorylation events more accurately. Additionally, given the advent of genetic mouse models to label endogenous proteins *in vivo* [e.g., biorthogonal noncanonical amino-acid tagging (BONCAT) (164)], coupled with new techniques that allow for kinome profiling (165, 166), future studies may also be able to identify endothelial PAR1/PAR4-specific kinases *in vivo* following receptor activation.

## Limitations

This review focuses on the understudied area of PAR4 on the endothelium. We have sought to contextualize our new findings with past reports about PAR4 functions on ECs and other cell types. We acknowledge that some sections of this review related to possible functions for PAR4 on ECs are postulative; these sections are labeled with headings in the form of questions. We intend for these sections to highlight gaps in the field of endothelial PAR4 biology and hope they inspire future studies from other labs.
